# Drug provocation tests: up-date and novel approaches

**DOI:** 10.1186/1710-1492-9-12

**Published:** 2013-04-03

**Authors:** Anca Mirela Chiriac, Pascal Demoly

**Affiliations:** 1 Allergy unit, Hôpital Arnaud de Villeneuve, 371 Avenue du Doyen Giraud, Montpellier, 34295, France; 2 INSERM U657, University Hospital of Montpellier, Montpellier Cedex 5, 34295, France

**Keywords:** Drug provocation test, Gold standard, Drug hypersensitivity reactions

## Abstract

**Summary:**

Drug provocation tests (DPTs) are often needed when evaluating patients with suspected drug hypersensitivity reactions. General considerations on DPTs, with regard to indications, contraindications, methods, limitations and interpretations have been thoroughly addressed and various protocols are published. However, the field of drug allergy is changing and DPTs make no exception. Novel (or sometimes, simply renewed) approaches arise, awaiting to be either validated or refuted in larger studies in the future. Instead of covering the whole topic of DPTs, this paper will address these recent and challenging aspects.

## Background

Drug provocation test (DPT) comes at the end of a step-wise approach in the drug allergy work-up. While there is general agreement that it is the procedure that holds the finest sensitivity amongst all the other available diagnostic tools, and that it may considerably improve patient management, its use as “gold standard” to establish (rather than just to exclude) the diagnosis of drug hypersensitivity reaction (DHR), is not unanimously accepted, due to the reactions it may trigger, depending on the severity of the index reaction.

Whether it is recommended by certain allergy societies [1] or guidelines [2] and briefly mentioned or debated by others [3], the decision of performing or not a DPT should only be taken on a patient to patient basis, after balancing the risk-benefit ratio in individual cases.

It cannot be disputed that DPTs represent a potential risk to the patient, are time consuming and need appropriate medical facilities and trained personnel. They are, however, sometimes the only resource available for confirmation or exclusion of hypersensitivity when previous investigation steps are negative, non-conclusive or simply unavailable [2,4]. General considerations on DPTs, with regard to indications, contraindications, methods, limitations and interpretations have been thoroughly addressed [2] and protocols published [5-8]. Nevertheless, the precise challenge procedure may vary a lot from one team to another and the need to standardize its practice has not been met so far.

On the other hand, data emerging from different centers, using different strategies of testing, from indication to interpretation of results, help move forward the field of drug hypersensitivity in terms of classification, diagnosis, impact on quality of life. Recent publications tackling DPTs have shown promising results, which await confirmation in the future. Instead of covering the whole topic of DPTs, this paper will address these recent and challenging aspects. For more detailed reviews on how to perform DPT, readers are referred to previously published guidelines and reviews [2,4,9,10].

### Methodology of DPT

Dosage of test preparations and time intervals vary between published studies. A maximum single dose of the specific drug must be achieved, and the administration of the defined daily dose is desirable. Depending on the type of drug itself, the severity of the DHR under investigation and its mechanisms, the expected time latency between application and reaction, DPT may take hours, days or, occasionally, weeks, before completion [2]*.* The evaluation of DHRs to beta-lactam antibiotics has raised the issue of resensitization after a negative allergological work-up (whether or not followed by full therapeutic courses), and concerns were expressed mainly in relation to immediate reactions [8]*.* Similarly, there’s a vivid controversy amongst different groups on whether one full therapeutic dose (of whatever tested drug) is sufficient enough to elicit reactions in non-immediate responders. Hence, full therapeutic courses have been suggested to increase the sensitivity of DPTs. However, such an indication is still under debate and must be regarded cautiously, in terms of diagnostic improvement, cost and medical implications. Hjortlund *et al.*[11] conducted a 7-day oral treatment for patients with an initial negative work-up (comprising IgE, skin tests and a single challenge protocol), according to the European Network for Drug Allergy (ENDA guidelines) [5,7]. 23 patients (*i.e.*, 5.7% of the 405 tested) were found to develop a reaction during the subsequent 7-day challenge with oral penicillin. Interestingly, the time interval to the occurrence of the clinical reaction in these patients was longer in most cases than that reported in the case history, which is in contrast to other studies in which it is shorter [12]. Another point worth mentioning is that intradermal tests were performed at lower concentrations than those recommended *(i.e.,* 1.25 mg/ml). Thus, whether or not some of these 23 patients might have actually displayed positive skin tests at higher, recommended concentrations, therefore being diagnosed by means of skin testing and not DTP, might be questioned.

### The scientific value associated to diagnostic DPT

DPT is considered the “gold standard” in the diagnosis of DHR to many drugs including nonsteroidal anti-inflammatory drugs (NSAIDs), due to the absence of reliable skin tests (or in-vitro tests), except for certain IgE mediated reactions. Because of a variety of clinical presentation of NSAIDs DHR (respiratory, cutaneous or anaphylaxis, and single or multiple reactors) [13], reliability of clinical history is difficult to assess, and most patients require DPT to confirm or refute diagnosis. The upmost interest of a DPT is to rule out the responsibility of a drug. Indeed, in our pioneer work [6] involving 1372 DPTs performed using various drugs, including ß-lactams (30.3%), aspirin (14.5%), other NSAIDs (11.7%), paracetamol (8.9%), macrolides (7.4%), and quinolones (2.4%), there were only 241 (17.6%) positive results. While being a diagnostic tool, confirming the role of NSAIDs in 13.4% [14] to 85.7% [15] of the suspicions, DPT holds an intrinsic scientific value, since it generates precious knowledge. In this respect, we [16] used data collected from a large database in 980 patients gathered over a 10-year period (using both the clinical history and the results of the DPTs) to put forward a new classification of NSAIDs DHR. The objective was to demonstrate whether or not patterns of NSAIDs reactivity are well defined with little or no overlap and to have the possibility of identifying patients at a high risk, with a rather high probability, according to clinical history and clinical manifestations. Based on our cohort of 122 (12.5%) positive patients, accounting for 307 positive DPTs and 105 negative challenges for finding an alternative drug, we were able to design a new classification, encompassing all our patients (some of which could not be included in the groups previously described by others) [13,14,17]. We chose to group the conditions comprising asthma/rhinosinusitis and/or urticaria/angioedema in one single entity of underlying diseases, because we noticed that the same clinical forms, *i.e.* immediate reactions (occurring immediately or up to 6 h after drug exposure) such as asthma/rhinitis and/or urticaria/angioedema can be seen with or without any underlying disease. Another major clinical innovation of our analysis is the highlighting of non-immediate angioedema (occurring between 6 to 24 h after exposure) as a form of non-immediate reaction to NSAIDs, which was not included in the previous classifications. This classification provides immediate application in clinical practice, since it offers an insight into the management of these patients, making it easier to decide which steps need to be followed to choose the drug to be tested (same drug, alternative drug from another NSAID family, or a COX-2 inhibitor directly).

### Peculiar aspects of drug allergy work-up in children

Studies comparing proven DHRs prevalence among different age populations are scarce and a recent study of ours [18] addresses this issue. We found a similar rate of proven DHRs in children and adult groups when anaphylaxis, anaphylactic shock, urticaria/angioedema were the suspected clinical manifestations (comprising 20%, 30% and 10% of the cases respectively). In maculopapular exanthemas, however, a remarkable difference of proven reactions was found between the two (3.1% in children vs. 12.8% in adults). This reinforces the fact that maculopapular exanthemas in children are not usually due to drugs and should clearly be differentiated from urticaria.

In children treated with beta-lactams, skin eruptions, mostly described as maculopapular or urticarial, are frequently assumed to be a drug-related allergy, although it has been suggested that most of these eruptions are actually not allergic in nature, with viral infections being often considered as the differential diagnosis, although the role of virus-drug specific T cell interactions still needs to be worked out. In a prospective study, Caubet *et al.*[19] conducted DPTs, irrespective of skin test results, in a population of 88 children with delayed-onset benign eruptions occurring during treatment with beta-lactam antibiotic. Based on the observation that none of the patients developed an immediate and/or a severe reaction during DPT, the authors suggest that a 1-dose DPT in children with a history of a delayed-onset benign reaction (assessed by a careful primary evaluation in the acute phase by an experienced allergist) can be considered safe. Although tempting, this approach needs validation in larger studies.

There is a general consensus amongst allergy societies about the contraindications of DPT, with respect to the severity of the initial reaction and the availability of immediate treatment allowing complete and fast recovery [2,3]. DPT should never be performed on patients who have experienced severe, life-threatening immunocytotoxic reactions, vasculitic syndromes, exfoliative dermatitis, erythema multiforme major/Stevens-Johnson syndrome, drug induced hypersensitivity reactions (with eosinophilia)/DRESS, toxic epidermal necrolysis or organ involvements. However, the US Practice Parameters [3] state that rare exceptions to this may exist, such as treatment of a life-threatening illness, in which case the benefit of treatment outweighs the risk of a potentially life-threatening reaction. In a large retrospective study regarding beta-lactam allergy in children, Ponvert *et al.*[20] suggest that serum-sickness like reactions, erythema multiforme, and Stevens-Johnson syndrome in children are mainly due to viral infections, and in such patients, with a negative allergological work-up based on non-immediate reading intradermal and patch tests, DPT might be considered for essential (future necessity) beta-lactams. However, this attitude must be regarded with the uttermost caution, since evidence supporting it is lacking and due to the high risk of recurrence of such reactions [21].

### DPT as a systematic diagnostic tool in non-immediate cutaneous hypersensitivity reactions to iodinated radio-contrast media (iRCM)

A recent European multicenter study [22] showed that patients with non-immediate iRCM allergy exhibit extensive skin tests cross-reactivity amongst different iRCM. Identification of a well-tolerated alternative iRCM is important once the allergy diagnosis has been established. However, the value of skin testing is limited in non-immediate reactors [23], as opposed to immediate reactors [24]. Based on their experience with the largest series of patients (161) published so far in non-immediate DHR to iRCM, undergoing a complete allergological work-up (including both skin and provocation tests), Torres *et al.*[12] recently suggested that DPT be used not only as a diagnostic tool in such patients, but also to certify tolerance of an alternative skin test-negative iRCM. Their data confirm that skin-test sensitivity in non- immediate reactors is insufficient, even using maximal concentrations (*ie*, intradermal test with undiluted iRCM), since more than half of the cases needed a DPT to be diagnosed. Moreover, in the group of patients with a positive skin test, tolerance could not be guaranteed to an alternative iRCM from a negative skin test, as DPT was positive in 32.3% of the patients.

### DPT as a diagnostic tool in the evaluation of perioperative anaphylaxis

DPTs have limited indications in perianesthetic anaphylaxis, since they are contraindicated with many anesthetics, such as muscle relaxants [25]. The guidelines on perioperative anaphylaxis of the French Society of Anesthesia and Resuscitation, endorsed by ENDA and the Drug Allergy Interest Group of the European Academy of Allergology and Clinical Immunology [25] recommend DPTs with drugs for which skin tests cannot be conducted or when skin tests are negative (local anesthetics, antibiotics) or not validated (NSAIDs, paracetamol). For general anesthetics, this gold standard allergy test cannot normally be used because of the pharmacological effects of these drugs. However, in the Danish Anesthesia Allergy Centre, such DPTs are extensively used. Nevertheless, some suspected drugs are only tested by administering up to maximum of one tenth of the therapeutic dose. This approach allows the diagnosis of DHRs such as dextrans (IgE-mediated mechanism) to be made and reduces the risk of both false positive test results (*i.e.*, to low-molecular-weight heparins) and false-negative test results (*i.e.*, to opioids). Muscle relaxants are not tested [26].

### Patient reported outcomes (PROs) in drug allergy

#### Questionnaires regarding satisfaction on DTP

The assessment of PROs has gained increasing importance over the last years, with investigators using different kind of surveys to obtain data collection. The drug allergy field makes no exception. The impact of diagnostic tools such as DPT has not been considered from the patient’s perspective until recently. 217 patients that underwent DPT for diagnostic purposes in 3 European centers were asked to fill in a questionnaire focused on the perceived advantages/disadvantages of the DPT and on the general satisfaction with the test. Data collected revealed that most patients accepted DPT for diagnostic purpose irrespective of the test final results, and 95% of them believed that it was useful and they would recommend it to others [27]. Similar findings were observed across centres, in adults as well as in children.

#### Negative predictive value (NPV) of DPTs

Though not completely established, the NPV of DPTs is important for both the patient and the physician. They both have to know whether a reaction can occur after taking a drug, which tested negatively. This information should reassure the patients and their doctors in prescribing drugs with negative DPT. However, even despite negative DPTs, a third of our patients were afraid of a potential reaction and did not take the negatively tested drug afterwards [28] and in a sixth of them, physicians refused to prescribe the negatively tested NSAID and preferred an alternative, as previously described with ß-lactam [29].

In spite of the advantages that DPT holds over all the other test procedures, it has its limitations. A negative test does not prove beyond any doubt tolerance for the drug in the future, but rather that there is no DHR at the time and conditions of the challenge. Indeed, IgE sensitivity may decrease over time [8], co-factors such as food, exercise, or viral infections may have been missed. Studies regarding the NPV of DPTs are encouraging. A high NPV of beta-lactam DPT of 94-98% was found in large studies involving 256 children [30] and 457 patients (both adults and children) [31] and most of the reactions reported by patients were mild non-immediate reactions. As for the antibiotics, the NPV of DPTs with NSAIDs appears to be also high (over 96%) whatever the NSAID (the one negatively tested or an alternative), and none of the false-negative patients described a life-threatening reaction [31]. The working hypothesis regarding our high NPV is that we finish our DPT with a high dose (as close to the 'cumulative daily dose' as possible) and this may be the reason why some recent studies [11], finishing their protocol with only 'one unit dose', have shown that extension of DPT protocol to one week leads to higher sensitivity.

## Conclusion

Whether the point of interest of the different groups working in the drug allergy field moves from the medical (indications/contraindications, results) to the technical (methodology) aspects of DPTs, or even addresses the patients perspective (PROs, satisfaction, NPV), the disclosure of such challenging and various results can only improve patient management. It remains to be seen which of these novel approaches will stand the test of time, and receive validation by larger studies.

## Competing interests

The authors declare that they have no competing interests.

## Authors’ contributions

Both authors read and approved the final manuscript.
